# Socioecological predictors of breastfeeding practices in rural eastern Ethiopia

**DOI:** 10.1186/s13006-022-00531-3

**Published:** 2022-12-28

**Authors:** Marina Magalhães, Amanda Ojeda, Karah Mechlowitz, Kaitlin Brittain, Jenna Daniel, Kedir Teji Roba, Jemal Yousuf Hassen, Mark J. Manary, Wondwossen A. Gebreyes, Arie H. Havelaar, Sarah L. McKune

**Affiliations:** 1 Department of Biobehavioral Nursing Science, College of Nursing, University of Florida, Gainesville, USA; 2 Present address: Department of Pediatrics, Stanford University School of Medicine, Stanford, CA USA; 3 Department of Animal Sciences, Institute of Food and Agricultural Sciences, University of Florida, Gainesville, USA; 4 Department of Microbiology and Cell Science, University of Florida, Gainesville, USA; 5 Department of Environmental and Global Health, College of Public Health, University of Florida, 1225 Center Dr., Gainesville, FL 32603 USA; 6 Department of Epidemiology, Rollins School of Public Health, Emory University, Atlanta, USA; 7 School of Nursing and Midwifery, College of Health and Medical Sciences, Haramaya University, Dire Dawa, Ethiopia; 8 Department of Rural Development and Agricultural Extension, College of Agriculture and Environmental Sciences, Haramaya University, Dire Dawa, Ethiopia; 9 Department of Pediatrics, School of Medicine, Washington University, St. Louis, USA; 10 Global One Health Initiative, The Ohio State University, Columbus, USA; 11 Emerging Pathogens Institute, University of Florida, Gainesville, USA; 12 Institute for Sustainable Food Systems, University of Florida, Gainesville, USA; 13 Center for African Studies, University of Florida, Gainesville, USA

**Keywords:** Breastfeeding, Infant feeding, Nutrition, Maternal child health, Ethiopia

## Abstract

**Background:**

Estimates by the World Health Organization indicate that over 800,000 global neonatal deaths each year are attributed to deviations from recommended best practices in infant feeding. Identifying factors promoting ideal breastfeeding practices may facilitate efforts to decrease neonatal and infant death rates and progress towards achieving the Sustainable Development Goals set for 2030. Though numerous studies have identified the benefits of breastfeeding in reducing the risk of childhood undernutrition, infection and illness, and mortality in low- and middle-income countries, no studies have explored predictors of breastfeeding practices in rural eastern Ethiopia, where undernutrition is widespread.

The aim of this study is to examine predictors of infant feeding practices in Haramaya, Ethiopia, using a multi-level conceptual framework.

**Methods:**

This study uses data collected from household questionnaires during the Campylobacter Genomics and Environmental Enteric Dysfunction (CAGED) project among 102 households in the Haramaya woreda, Eastern Hararghe Zone, Eastern Ethiopia, and investigates factors influencing breastfeeding practices: early initiation, prelacteal feeding, and untimely complementary feeding.

**Results:**

Nearly half (47.9%) of infants in this study were non-exclusively breastfed (*n* = 96). Generalized liner mixed effects models of breastfeeding practices revealed that prelacteal feeding may be a common practice in the region (43.9%, *n* = 98) and characterized by gender differences (*p* = .03). No factors evaluated were statistically significantly predictive of early initiation and untimely complementary feeding (82% and 14%, respectively). Severely food insecure mothers had more than 72% lower odds of early breastfeeding initiation, and participants who self-reported as being illiterate had 1.53 times greater odds of untimely complementary feeding (95% CI, [0.30,7.69]) followed by male children having 1.45 greater odds of being untimely complementary fed compared to female (95% CI,[0.40,5.37]).

**Conclusions:**

This study found high rates of prelacteal feeding and low prevalence of exclusive breastfeeding, with girls more likely to be exclusively breastfed. While no predictors evaluated in this multi-level framework were associated with prevalence of early initiation or complementary feeding, rates may be clinically meaningful in a region burdened by undernutrition. Findings raise questions about gendered breastfeeding norms, the under-examined role of khat consumption on infant feeding, and the complex factors that affect breastfeeding practices in this region. This information may be used to guide future research questions and inform intervention strategies.

**Supplementary Information:**

The online version contains supplementary material available at 10.1186/s13006-022-00531-3.

## Background

Promoting and protecting breastfeeding is a global health imperative. The WHO estimates that breastfeeding can prevent 820,000 child deaths per year, as well as nearly half of diarrhea episodes and one third of respiratory infections [1]. UNICEF has emphasized that investing in and improving breastfeeding practices, including early initiation, duration, and exclusivity, is fundamental to achieving the Sustainable Development Goals of ending poverty and hunger, reducing undernutrition and child mortality, and improving education and economic growth [2]. However, barriers to optimal breastfeeding pervade at all social-ecological levels, requiring an exploration of their contextual relationships to inform future breastfeeding interventions [3].

This study uses data collected during the formative research of the Campylobacter Genomics and Environmental Enteric Dysfunction (CAGED) project, which investigated the burden of *Campylobacter* colonization, environmental enteric dysfunction, and impaired growth in children in Haramaya woreda district, East Hararghe Zone, Oromia Region, Ethiopia. Early ethnographic research from the study revealed that 53% of infants displayed visible symptoms of undernutrition [4] and additional formative research found that 41% of children between 12 and 15 months of age were stunted [5]. Breastfeeding is an important confounder in the CAGED project, whose overall objective is to improve child nutrition by reducing exposure to zoonotic pathogens from livestock.

Undernutrition lowers the body's ability to resist infection by undermining the functioning of the main immune-response mechanisms, leading to longer, more severe, and more frequent episodes of illness. Meanwhile, breastfeeding lowers the risk for contracting infectious, respiratory, and diarrheal diseases [6, 7] and decreases the overall risk of infant morbidity and mortality [8]. In Ethiopia, 39% of children under 5 years are stunted [9], while only 47% of infants are exclusively breastfed until 6 months of age [10]. Given the important role of breastfeeding in child nutrition outcomes, optimizing breastfeeding practices in Ethiopia could have important positive health implications for children amid widespread undernutrition. A more thorough investigation into the CAGED formative research dataset is therefore warranted to better understand factors predictive of breastfeeding practices. The aim of this study is to examine predictors of infant feeding practices in Haramaya, Ethiopia, using a multi-level conceptual framework. The work relies heavily on the socioecological model used in public health. While the model and its development are explained in the methods section, we first present the literature on factors associated with breastfeeding practices by levels derived from our model. These levels include 1) basic, 2) underlying, 3) immediate, and 4) physiological factors.

### Basic predictors

Our model begins with *basic predictors* of breastfeeding practices, including the policy, climate, and economic context. Agriculture is the backbone of the Ethiopian economy, employing an estimated 75% of the population. Cash cropping has emerged as a major player in improving the livelihoods of Ethiopian smallholder farming households, which includes khat, a cathinone-releasing stimulant and one of the largest sources of internal tax revenue. [11, 12]. The increased income from khat production combined with access to and participation in markets may contribute positively to food security and dietary diversity among some households [12, 13]. In this area, khat production is a gendered practice and tends to be grown to be sold at the market largely by women, raising questions about the role of khat production in the health and behaviors of women and children [14, 15].

### Underlying predictors

The second level in our model is underlying predictors, including education, culture, religion, and empowerment. Findings on maternal age in Ethiopia suggest suboptimal breastfeeding among women of the youngest childbearing ages (15 to 24 years), with higher rates of early breastfeeding initiation and exclusive breastfeeding (EBF) among mothers aged 25 to 35, and earlier termination of breastfeeding among women younger than 25 [10, 16, 17].

Women’s empowerment is integral to the Sustainable Development Goals and is associated with reduced odds of child growth faltering in Ethiopia [18]. Empowerment can allow women more decision-making concerning household purchases, healthcare access, their reproductive health, and ability to provide Essential Newborn Care, including breastfeeding [19–21]. Only 48% of women in rural Ethiopia have attended any formal school [22]; those with formal education are more likely to engage in optimal breastfeeding practices [23].

Cultural norms are known to strongly influence breastfeeding practices. In Ethiopia, studies have explored cultural underpinnings of common practices. Concerns regarding abdominal cleansing and milk insufficiency may promote prelacteal feeding and early complementary feeding [24]. Orthodox Christianity (43%) and Islam (31%) are the two major religions in Ethiopia [22] and both involve periods of fasting throughout the year, although practices during pregnancy and postpartum vary widely [25]. Fasting during pregnancy or lactation may influence maternal nutritional status and child feeding practices [26, 27].

### Immediate predictors

The third level in our model is immediate predictors. Maternal access to a health facility and care are strong predictors of EBF and timely initiation of feeding [10, 16]. Consistent with other LMIC [28], employment has been found to be a predictor of exclusive breastfeeding in northwest Ethiopia [29]. Most self-employed women in this region work as farmers and are more likely to exclusively breastfeed than women who are employed outside the home, while unemployed mothers are more likely to exclusively breastfeed than those employed [29]. Mothers who work outside the home may have limited time or space to breastfeed at work.

Fathers play a key role in child health and wellbeing, and paternal support has been linked with prolonged EBF in Ethiopia [30, 31]. A systematic review of breastfeeding interventions found that support from fathers significantly affected mothers' intention to exclusively breastfeed across LMIC [32].

### Physiological predictors

The final level in our model includes predictors which may physiologically influence the amount of milk available for infant consumption. One of the most frequently cited reasons for short lactation or complementary feeding in both high and LMIC is insufficient milk supply [33]. Breast milk volume and duration are dependent on a positive feedback cycle and may be supported or impeded by several factors, such as timely initiation of breastfeeding [34]. A meta-analysis in Ethiopia found that infants who received prelacteal foods were 28% less likely to receive timely breast milk, likely shortening their breastfeeding duration [35]. Additionally, proper positioning and infant latch are critical for preventing pain and optimizing milk transfer; a 2018 study found the prevalence of optimal breastfeeding technique to be just 43% in eastern Ethiopia [23].

The importance of frequent feeding for milk supply and neonatal weight gain [36] highlights the need for research on whether the stimulating or appetite-suppressing effects of khat [37] play a role in breastfeeding practices. While khat use is not included in our conceptual model due to limited existing literature on its use during lactation, 1 in 5 pregnant women in one multi-center study in Ethiopia reported to chew khat most days per week [38]. Norpseudoephedrine, the active substance from khat, has been detected in breast milk among consumers [39] and is associated with gastrointestinal disorders and decreased nutrient absorption among consumers [40]. Given the intensity of khat production and consumption in and around Haramaya woreda, consideration of how khat production (including farming and sale/exchange) and consumption may affect breastfeeding frequency and duration may be important.

## Methods

### Breastfeeding socioecological model

**Fig. 1:**
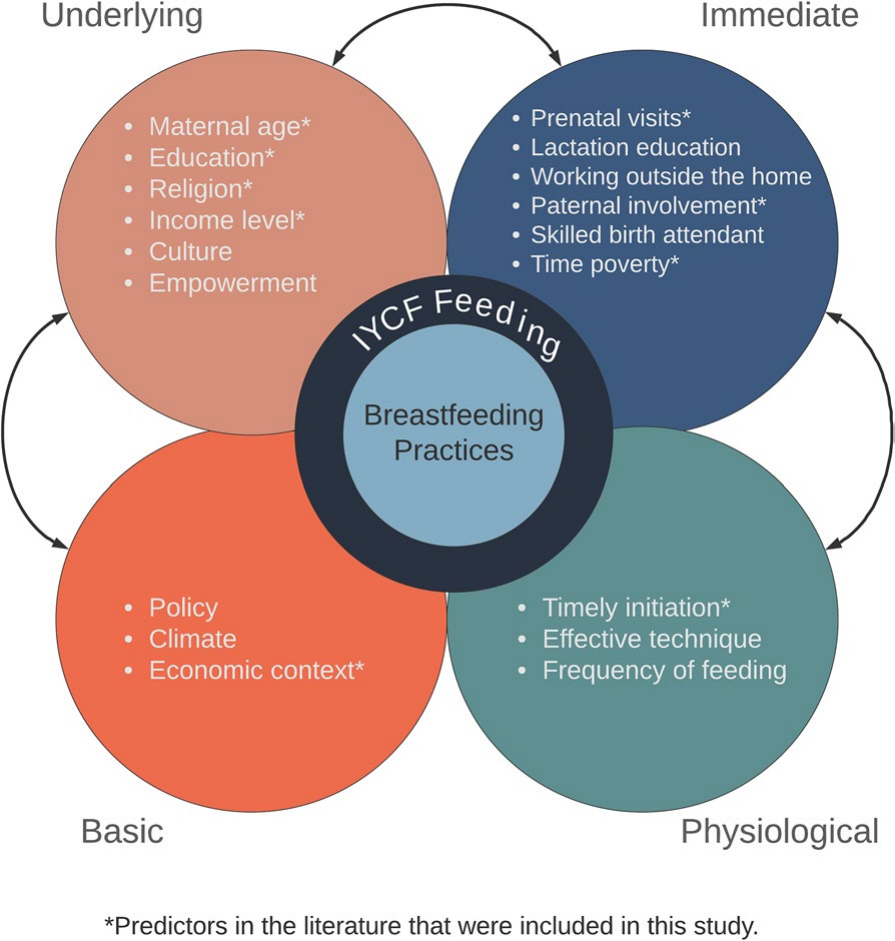
Socioecological predictors of breastfeeding practices and infant and young child feeding

Our breastfeeding practices model was developed as a conceptual framework that merges the socioecological model [41] with UNICEF's conceptual framework on the causes of undernutrition [42]. As described in the introduction, the UNICEF model was adopted and modified to understand: 1) basic, 2) underlying, 3) immediate, and 4) physiological factors that impact breastfeeding practices in Haramaya. In the UNICEF model, suboptimal breastfeeding falls under “care practices” and is an underlying cause of undernutrition. Our model (Fig. 1) integrates these undernutrition factors with breastfeeding practices as our outcomes of interest. Further, our model incorporates factors at the physiological level which have been described in the literature to impact breastfeeding. While the model is certainly not exhaustive, it serves as a framework for conceptualizing how multilevel factors such as economic context, empowerment, and delayed breastfeeding initiation may affect breastfeeding practices in the study population. Breastfeeding practices in Haramaya are largely unknown and thus factors in each level of the model are derived from current literature on predictors of infant feeding in other regions, including in Ethiopia.

At the center of our conceptual model is breastfeeding practices. These practices include early initiation, prelacteal feeding, colostrum avoidance, exclusive breastfeeding, and untimely complementary feeding. These practices are determined based on recommended and discouraged infant feeding practices reported in the literature. Optimum feeding practices include breastfeeding immediately after birth (early initiation), offering no other foods or liquids (exclusive breastfeeding), and initiating complementary foods no earlier than 6 months of age. The WHO also discourages introducing complementary foods later than 6–8 months of age, as breast milk is then insufficient for the nutritional needs of the infant. Prelacteal feeding is also a discouraged practice, defined as offering food or drinks (such as water, honey, formula milk, or fruit juice) to a newborn prior to establishing breastfeeding.

### Overview

CAGED formative research included rapid ethnography, full household enumeration within the study region, household questionnaires, anthropometric measurement of children, and collection of samples for laboratory analysis. The primary aims, research questions, and results have been published elsewhere [4, 5, 43]. Using data collected through household questionnaires during the formative research, this study examines breastfeeding practices in Haramaya woreda. Full details of data collection methodology have been previously published [5] but are presented briefly below for ease of reference.

### Study site and population

Haramaya woreda is a semi-arid region of East Hararghe Zone, Oromia Region, where most of the population practices mixed crop-livestock livelihoods. High rates of extreme poverty burden the region, and women are predominantly responsible for both infant and livestock housing, safeguarding, feeding, and healthcare [4]. It is a district characterized by large family sizes, high fertility rates, and very high childhood undernutrition rates [5]. The region is highly affected by climate change, increasing population pressure, and reducing land and water availability. Khat production is pervasive and has partially or fully replaced grain and vegetable production in many households across the region [12]. Within this context, households were targeted for inclusion in the study (see details below); while men, women, and children were participants in the overall study [5] data included in analysis here all come from surveys conducted with women in those households.

### Study design and sample

In Ethiopia, *kebeles* are the smallest geographic administrative units recognized in census mapping. Five of the twelve Haramaya kebeles were selected for inclusion in the formative research based on maximized geographical distance between the kebeles. Household surveys were conducted with men and women in randomly targeted households across the five Haramaya kebeles. Eligibility criteria of the household included the presence of at least three chickens in the homestead (defined as a collection of households, often extended family, that share common areas), non-participation in any other animal husbandry project, and having lived in Haramaya woreda for at least three months. Eligibility criteria of the child included the absence of any visible congenital anomalies, absence of an extended hospital stay for mother or child following the child's birth, and child age of 11–13 months. A total of 102 households were randomly selected and completed the household survey. Data were collected between September and December 2018. The sample size was based on the primary research objectives of the formative research; thus, this study is not powered to measure maternal and infant health or breastfeeding outcomes.

Research presented here is covered under the CAGED study protocol that underwent ethical review and received approval from the Haramaya University Institutional Health Ethics Research Review Committee (IHRERC/152/2018), the Ethiopia National Research Ethics Review Committee (MoST/3–10/168/2018), and the Institutional Review Board at the University of Florida (201,703,252).

### Survey development

The household survey was developed in collaboration with the University of Florida (UF) and Haramaya University (HU) teams. A draft was developed initially by the UF team, then shared electronically with the HU team, who made substantive revisions in early 2018. The modified version was entered in April/May of 2018 into REDCap electronic data capture tools hosted at University of Florida Clinical and Translational Science Institute [44]. REDCap draft survey was loaded onto Samsung Galaxy Tablets, where all data entry was conducted. Substantial revisions came from a three-step piloting and revision process that occurred during a two-week training workshop at Haramaya University in August 2018. First, three Ethiopian data collectors recommended a round of revisions after reading the tablet-based survey independently and with each other. A second round of revisions was provided by an extended team of social science and public health experts from UF and HU who went over the survey together, line by line. The team took two full days to review the survey, validate local examples, identify appropriate food choices, brand names, and vitamins available, and reword or remove questions that were not culturally appropriate or relevant. The team also discussed at length how best to ask each question, including what translation into Afan Oromo would be most appropriate to preserve the meaning of questions. Finally, the three data collectors and supervisor field-tested the survey within local non-eligible communities near Haramaya University. Revisions were made to the final survey.

### Variables

The primary outcome variables for this study were 1) early breastfeeding initiation, 2) prelacteal feeding, and 3) untimely complementary feeding. Early initiation was coded dichotomously as whether the infant was put to the breast within one hour of birth. Prelacteal feeding was coded as a dichotomous variable, defined by whether the child was given anything to drink other than breast milk during the first 3 days after delivery. Untimely complementary feeding was measured by identifying the child’s age when first given something to eat other than breast milk (either early – before 6 months or late – after 8 months). Exclusive breastfeeding was analyzed only descriptively, as this practice is a composite of other breastfeeding practices in the analysis but is defined as the infant receiving breast milk and nothing else for the first 6 months of life. Several standard predictor variables used in this study (ie. maternal age, and number of antenatal care visits) were generated using single questions from the household survey. However, composite variables were also used; further detail on methods used to generate those variables is included below.

Time poverty was monitored using the Women’s Empowerment in Agriculture (WEIA) Index. Participants are asked to define number of hours (sum amount of time) of work-related tasks (ie. employment, own business work, farming, cooking) performed 24 h prior to a household interview [45]. Individuals who worked more than 10.5 h in the day were identified as time impoverished [45]. Whether the participant’s primary source of income was from khat was measured by a single question in which the participant was asked to identify their primary source of livelihood (animal production, crop production, khat production, petty trade, remittances, or other). Severe food insecurity was measured using the Household Food Insecurity Access Scale (HFIAS) [46] with items which asked whether the participant experienced at least one of these *often* (more than ten times in the past month): running out of food, going to bed hungry, or going a whole day and night without eating. Food secure, mildly food insecure, and moderately insecure households were not identifiable as our questionnaire included a subset of questions for severe food insecurity from the HFIAS.

### Statistical analysis

Statistical analysis was conducted using R version 4.0.2. Descriptive statistics were conducted to characterize breastfeeding practices, potential covariates, and additional factors such as food insecurity. Bivariate logistic regression was conducted on our breastfeeding outcomes. Untimely complementary feeding was disaggregated to early and late complementary feeding in our bivariate analysis and aggregated for multivariate models. Predictors that met the established cut-off (*p*-value < 0.20) (Table 2) were included in the multivariate analysis. Given the sample size, a backward selection approach was taken to conduct generalized linear mixed effects modeling (GLMM) to account for any dependency between clustered covariates and response variables. The lme4 package in R was utilized for GLMM analysis. Models were created to understand the effect of potential confounders (ie. mother’s age, literacy, child sex, kebele) on each outcome. To select the best fitting model and assign covariates as a fixed or random effect, variance, Akaike information criterion, Bayesian information criterion and log likelihood scores were evaluated. Mother’s age, literacy, and child sex were considered as fixed effects while kebele was included as a random effect in all models. Odds ratios were calculated with a 95% confidence interval to determine the relative odds of targeted breastfeeding practices occurring given exposure to variables of interest.

## Results

The results represent 102 households residing across five kebeles in rural eastern Ethiopia. The number of households from each kebele was proportional to the population of each kebele, with 30.3% of households from Kebele 1, 10.8%, from Kebele 2, 19.6%, from Kebele 3, 9.8% from Kebele 4, and 29.4% from Kebele 5. Most households indicated that khat production was their main form of livelihood; 93.5% from Kebele 1, 81.1%, from Kebele 2, 90% from Kebele 3, and 86.6% from Kebele 5. Kebele 4's livelihood stemmed mainly from crop production (80%).

Mothers' ages ranged between 16 to 37 years, with the median age being 25 years. Nearly 3 of 4 women (73.7%) were illiterate as defined by self-reported inability to read or write. Further details on the factors at the underlying level can be found in Table 1.

**Table 1:** Bivariate analysis of demographic variables (*n* = 102)

Variables of Interest	Total (%)	Early Initiation *p-*value	Prelacteal Feeding *p-*value	Untimely Complementary Feeding	
				Early *p-*value	Late *p-*value
Underlying Factors					
Mother's Age (*N* = 101)		1	0.84	0. 16*	1
< 25 (16–24)	42.57				
> 25 (25–40)	57.43				
Mother's Literacy (*N* = 99)					
Literate	26.3	1	0.64	0.24	0.32
Illiterate	73.7				
Khat Use (*N* = 102)		0.30	0.68	0.03*	1
Daily Past Month	67.7				
Immediate Factors					
Antenatal Care Visits (*N* = 102)		0.25	0.37	1	0.32
> 4 visits	26.5				
< 4 visits	37.3				
0 visits	36.2				
Time Impoverished (*N* = 102)	53.9	0.80	0.69	0.50	0.37
Father living in Household (*N* = 102)	96	0.15*	0.32	0.32	1
No. living in household with the child (*N* = 102)					
> 6 individuals	48	0.80	0.16*	0.16*	0.67
Physiological Factors					
No Factors					
Basic Factors					
Food Insecurity, Severe (N = 102)	31.4	0.00*	0.51	0.72	0.64
Kebele		0.00*	0.01*	0.77	0.19*
1	30.3				
2	10.8				
3	19.6				
4	9.8				
5	29.4				
Primary income from khat (*N* = 102)	81.3	0.18*	0.44	0.20*	1
Additional Factors^1^					
Iron Suppl. (Any) (*N* = 100)	48	0.43	1	0.72	0.05*
Drugs for Intestinal Worms (*N* = 101)	1	1	1	0.09*	1
Anti-Malaria Medication (*N* = 101)	2	1	0.18*	1	1
Bed Nets Used Daily (*N* = 101)	24.7	0.55	1	0.45	0.33
Child Sex (*N* = 102)		0.30	0.02*	0.03*	0.36
Male	50				
Female	52				
Access to Water Distance (*N* = 99)		0.98	0.96	0.68	0.39
In the home	3.03				
Less than 5 min away	9.09				
Less than 30 min away	45.45				
Less than 1 h away	20.2				
More than 1 h away	22.2				

^1^Additional Factors are those included in our bivariate analysis but not in our conceptual model

^2^Covariates in our conceptual model meeting the *p* < .2 threshold were included in regression models

At the immediate level of our model, nearly 3 in 4 women (73.5%) attended fewer than four antenatal visits, including 36.2% of women who received no antenatal care. Based on the 24-h recall responses, 53.9% of mothers reported experiencing time poverty, of which 7.84% (*n* = 8) indicated they participated in non-farm economic activities including running a small business or self-employment in the previous 12 months. Nearly 1 in 3 women (31.4%) frequently experienced severe food insecurity, as assessed using the Household Food Insecurity Access Scale (57), and more than half reported to have chewed khat daily over the past month.

**Fig. 2:**
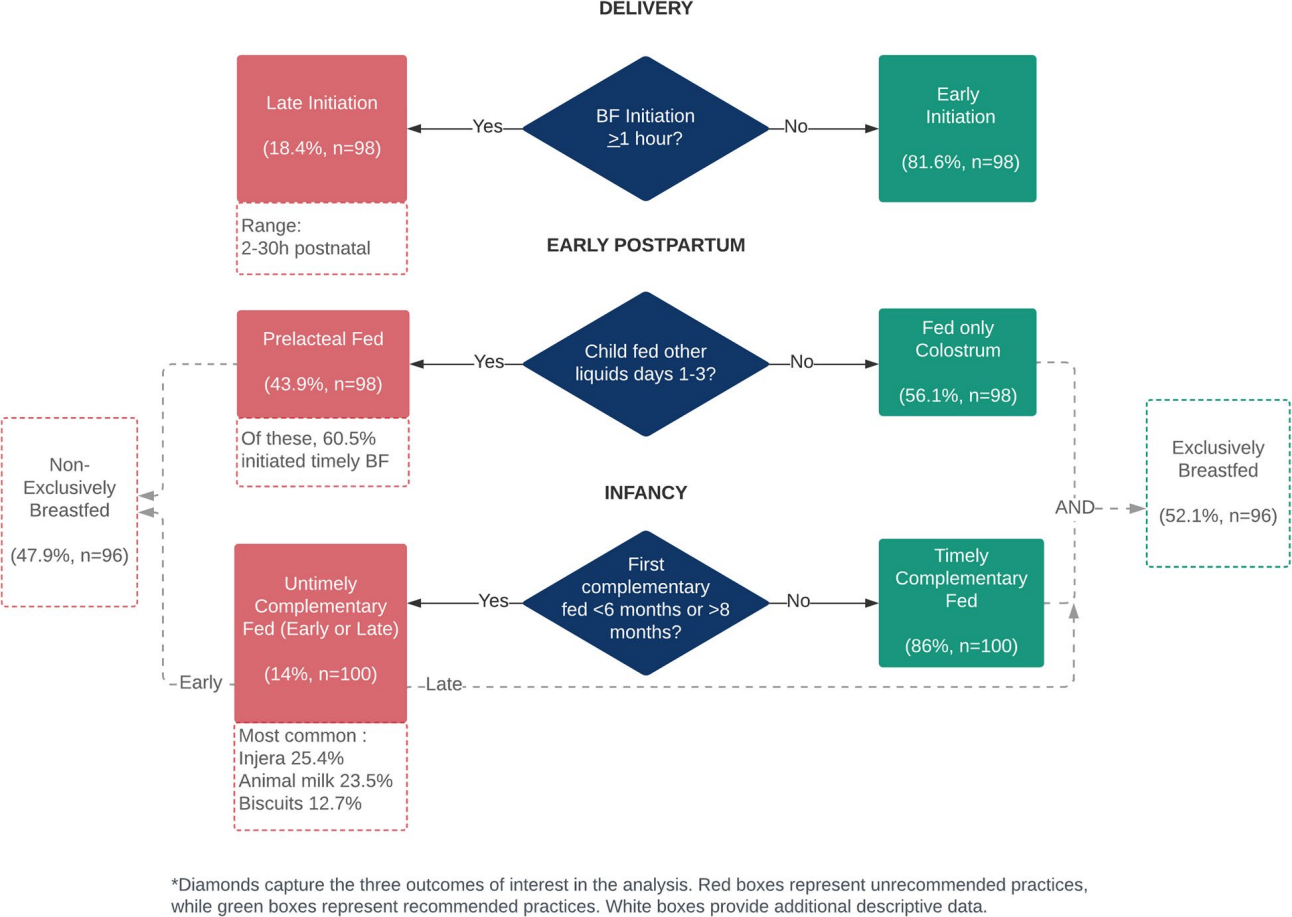
Diagram of breastfeeding practices and their relationships in Haramaya, Ethiopia

Of the 102 mothers, 81.6% breastfed their child within one hour of birth and 43.9% indicated having prelacteal fed their child (Fig. 2). Fourteen percent of participants introduced complementary foods in an untimely manner–9% early (before 6 months of age), and 5% late (beyond age of 8 months). Infants complementary fed early were predominately male (88.9%, *n* = 8). The most common foods first introduced were injera (Ethiopian flatbread) (25.4%, *n* = 26), animal milk (23.5%, *n* = 24), and biscuits (12.7%, *n* = 13). Additional descriptive statistics on breastfeeding practices can be found in the Supplementary materials.

Following backward selection, child sex, number of people sleeping in the same room as the child, and kebele met the required threshold for inclusion in the model for prelacteal feeding. Variables preserved in the model for untimely complementary feeding were consumption of khat, consumption of iron supplements, bed net use daily, and number of people sleeping in the same room as the child. Kebele, severe food insecurity, primary income from khat, and the father’s residence in the household were included in the model for early breastfeeding initiation. Maternal use of bed nets daily, preventative malaria medication, or drugs for intestinal worms during pregnancy was excluded following backward selection. Mixed effect modeling revealed that mothers whose household income was dependent on khat had 91% lower odds of early breastfeeding initiation (*p* = 0.12), and severely food insecure mothers had more than 72% lower odds of early initiation (*p* = 0.13). Mothers who were illiterate (*p* = 0.37) and those whose infant’s father was living in the household (*p* = 0.57) both had more than twice the odds of early breastfeeding initiation (Table 2). Further, mothers who self-reported as being illiterate had 1.5 times the odds of practicing untimely complementary feeding (*p* = 0.61), and older mothers ( between 25–40 years of age) had greater odds (1.4) of practicing untimely complementary feeding (*p* = 0.70). Male infants had 2.74 times the odds of being prelacteal fed (*p* = 0.03) and 1.45 time the odds of being untimely complementarily fed compared to female infants (*p* = 0.58) (Tables 3 and 4).

**Table 2:** Early initiation of breastfeeding mixed model regression summary (*n* = 94)

	Estimate	Std. Error	z value	Pr( >|z|)	OR	95% CI
Child sex (males)	-0.74	0.72	-1.03	0.30	0.48	(0.12, 1.95)
Mother’s age (≥ 25yrs)	-0.35	0.73	-0.48	0.63	0.71	(0.17, 2.96)
Mother illiterate	0.87	0.96	0.90	0.37	2.39	(0.36, 15.78)
Income dependent on khat	-2.41	1.53	-1.58	0.12	0.09	(0.00, 1.80)
Father living in household	0.91	1.59	0.57	0.57	2.49	(0.11, 56.38)
Severely food insecure	-1.27	0.84	-1.52	0.13	0.28	(0.05, 1.44)

^1^Kebele included as a random effect

**Table 3:** Prelacteal feeding mixed model regression summary (*n* = 93)

	Estimate	Std. Error	Z Value	Pr( >|Z|)	OR	95% CI
Mother illiterate	-0.09	0.59	-0.15	0.88	0.91	(0.29, 2.91)
Child sex (males)	1.01	0.47	2.14	0.03	2.74	(1.09, 6.89)
Mother’s age (≥ 25yrs)	0.98	0.66	1.48	0.14	2.67	(0.75, 9.82)
No. living in household with child	-1.27	0.66	-1.92	0.06	0.28	(0.08, 1.03)

^1^Kebele included as a random effect

**Table 4:** Untimely complementary feeding mixed model regression summary (*n* = 92)

	Estimate	Std. Error	Z Value	Pr( >|Z|)	OR	95% CI
Mother illiterate	0.42	0.83	0.51	0.61	1.53	(0.30,7.69)
Child sex (males)	0.37	0.67	0.56	0.58	1.45	(0.40,5.37)
Mother’s age (> 25yrs)	0.34	0.88	0.39	0.70	1.40	(0.25,7.79)
Khat consumption	-1.19	0.84	-1.41	0.16	0.31	(0.06,1.59)
Consumption of iron supplements	-1.73	0.83	-2.07	0.04	0.18	(0.04,0.92)

^1^Kebele included as a random effect

## Discussion

This study explores factors associated with breastfeeding best practices in Haramaya, Ethiopia. While the exclusive breastfeeding rate was 52%, this prevalence is higher than Ethiopia’s national average (47%) and infants globally (44%). The majority (81.6%) of infants were breastfed within one hour of birth, yet the prevalence of suboptimal breastfeeding practices in this region was largely driven by prelacteal feeding. Prelacteal foods are deficient in the nutritional and immunoprotective components of breast milk and increase risk for disease, stunting, and the long-term consequences of undernutrition [27]. Although colostrum feeding was not measured in this study, prelacteal feeding often coincides with colostrum avoidance and delayed breastfeeding initiation in LMIC, including Ethiopia [30], posing an increased risk for child morbidity and mortality [28]. However, our finding that over 60% of prelacteal fed infants were also breastfed within one hour of birth suggests that some infants may be receiving colostrum along with prelacteal foods.

Prelacteal feeding in this region (43.9%) remains far more commonplace than the 25.3% national average but remains in the middle range compared to other woredas in the Oromia region, which range from 5.9% to 75.8% [35]. When including infants who received complementary foods before the age of 6 months, nearly half of infants in this study were non-exclusively breastfed.

Interestingly, mothers who are illiterate had 2.39 times greater odds of initiating breastfeeding within the recommended first postpartum hour than were literate mothers. This contradicts a study in the nearby urban Harari region of Ethiopia which found that women with formal education were more likely to breastfeed immediately following delivery [23]. Delayed breastfeeding initiation ranged from 2 to 30 h postpartum, likely increasing risk for short breastfeeding duration [30].

We found that sons had 2.74 times greater odds of receiving prelacteal foods and half the odds of early breastfeeding initiation compared to daughters. These findings are similar to a national DHS study in Ethiopia which found male sex to be correlated with late breastfeeding initiation [10]. In contrast, one meta-analysis of observational studies found that mothers with sons had greater odds of exclusive breastfeeding [47]. These gendered findings reflect that infant feeding practices are embedded in sociocultural influences, some of which may manifest in gendered practices. Son preference has been widely explored in many countries in the context of birth spacing and fertility, and studies in Ethiopia have consistently found shorter pregnancy intervals following the birth of daughters than of sons [48]. Gendered perceptions about infant needs for growth, insufficient milk supply, infant digestive health, and protection against illness may promote prelacteal and early complementary feeding [24, 34]. However, to our knowledge, no studies in Ethiopia have explored sociocultural norms regarding infant sex and prelacteal feeding or complementary feeding.

To the authors' knowledge, this is the first study in Eastern Ethiopia that includes both early and late cessation of EBF, as most studies examine only early cessation. Early cessation of EBF is associated with greater incidence of diarrhea, fever, and acute respiratory infections [49]. Meanwhile, delayed introduction of nutritious complementary foods can lead to undernutrition and stunted growth, as breastmilk becomes insufficient to meet nutritional needs beyond 6 months [50]. The most commonly identified foods first initiated in this study were biscuits, cattle milk, and injera. While incidence of late complementary feeding is 5% in our sample, the vast majority of first foods introduced offer low complementary nutritional value, and our threshold of 8 months for initiation of complementary foods is liberal compared to the WHO recommendation of 6 months. While underpowered to detect statistical significance, the finding that one in twenty infants had not yet received any additional foods at 8 months of age may be of clinical interest for child growth and development. As over half of children in the CAGED ethnographic study were found to have visible symptoms of undernutrition, examining prelacteal feeding and complementary feeding more closely may be valuable to understanding growth faltering in the region. Discrepancies in feeding behavior based on child sex suggest the need for research to understand and address the gendered dynamics of breastfeeding practices.

### Strengths and limitations

As the parent study of this research was powered to different outcomes of interest, the objective of this study was not to detect for significance or generalize findings to the population, but to identify patterns of breastfeeding practices within this study population to inform future research and interventions in the area. Survey questions were developed based on aims for the CAGED formative research study; thus, the sample was not powered for our study outcomes and data available did not include every indicator in the conceptual framework, notably obstetrical and care indicators such as delivery by a skilled birth attendant and lactation education [51]. Since data were collected as reported from the mother–either by self-report or for the child–there is risk of recall or social desirability bias.

A major strength of this study is its design, sampling methodology, and survey development. Households were selected randomly and proportionately to their populations in each kebele. The household survey underwent extensive development, revisions, and piloting with collaborators at Haramaya University, including for validity, relevance, translation, and cultural sensitivity, and is based on ethnographic research that was done first. Additionally, this study is distinct in its reliance on a multi-level conceptual framework as there is a dearth of literature exploring how factors at multiple levels influence breastfeeding practices. Several factors included in this study have been poorly examined regarding breastfeeding practices, including delayed initiation of complementary foods, time poverty, khat use, food insecurity, household income and livelihood, and residence.

### Future research

This study reinforces the need for more in-depth investigation of factors and how they interact to effect breastfeeding practices. A larger sample size, powered around the variability of breastfeeding outcomes, is necessary. For instance, given the high prevalence of severe food insecurity in the study region, it may be more meaningful to further disaggregate women by the degree of severity experienced. Further, dependence on khat for household income had increased odds for suboptimal breastfeeding practices, and nearly 68% of women self-identified as daily khat consumers, yet there is minimal literature on its clinical relevance during lactation. Given the appetite-suppressing effects of khat, examining its consumption with feeding frequency and infant growth is needed in this region.

Qualitative research is needed to elucidate perceptions or practices that may be driving the gendered differences in infant feeding. Notably, male sex is associated with stunting among infants of malnourished women in Ethiopia [9]. However, male sex has been documented to be associated with stunting across 35 countries in sub-Saharan Africa [52]. It remains unknown whether this phenomenon is driven by biological underpinnings.

This population was identified to have low levels of literacy, and one in three women received no antenatal care; only a quarter (26.5%) received the recommended four visits. The high prevalence of prelacteal feeding in this region suggests that perinatal health education and breastfeeding support may be lacking, such as lactation education, paternal or familial engagement, or skin-to-skin care immediately following delivery. Qualitative research may elucidate mothers’ perspectives or barriers to receiving perinatal care and guide culturally informed community-based infant feeding interventions. As feeding practices in this region of Ethiopia are starkly different from other regions in the country [35], a geospatial analysis may further examine factors such as infrastructure or resource boundaries driving feeding outcomes.

## Conclusions

Early infancy is a unique window of opportunity for preventing illness and maximizing lifelong potential. In Haramaya woreda, a region of Ethiopia with 41% prevalence of child stunting at 12–15 months of age, prelacteal feeding is a common practice associated with infant sex. It is the authors' intention that the findings of this study inform future research studies that pave the way for targeted, culturally appropriate interventions to optimize infant breastfeeding practices and mediate in the cycle of undernutrition.

## Supplementary Information

**Additional file 1:Table S.** Breastfeeding practices summary statistics in Haramaya, Ethiopia.

## Data Availability

The datasets used and/or analysed during the current study are available from the corresponding author on reasonable request.

## Abbreviations

CAGED: Campylobacter genomics and environmental enteric dysfunction
EBF: Exclusive breastfeeding
GLMM: Generalized linear mixed effects model
HU: Haramaya university
IYCF: Infant and young child feeding
LMIC: Low- and middle-income countries
UF: University of Florida
WHO: World health organization
