# Supplementary material for: Socioecological predictors of breastfeeding practices in rural eastern Ethiopia
Source: Int Breastfeed J. 2022 Dec 28;17:93. doi: 10.1186/s13006-022-00531-3 (PMC9795740; doi:10.1186/s13006-022-00531-3)
Supplement: Supplementary file 1 — Additional file 1:Table S. Breastfeeding practices summary statistics in Haramaya, Ethiopia. [file 13006_2022_531_MOESM1_ESM.docx]

Supplementary Materials

Table S. Breastfeeding practices summary statistics in Haramaya, Ethiopia

| Breastfeeding Practices | n (%) |
| --- | --- |
| Early Initiation of Breastfeeding (N=98) | |
| Breastfed within one hour of birth | 80 (81.6%) |
| Prelacteal Feeding (N=98) | |
| Offered infant food or drinks during first 3 days postnatal | 43 (43.9%) |
| Exclusive Breastfeeding (N=96) | |
| 6 months | 50 (52.1%) |
| Breastfeeding Duration (N=100) | |
| Breastfeeding at time of survey (360-498 days old) | 90 (90%) |
| Complementary Feeding (N=100) | |
| Timely – between ages 6-8 months | 86 (86%) |
| Untimely Early – before 6 months | 9 (9%) |
| Untimely Late – after 8 months | 5 (5%) |
| Foods First Fed (N=102)  Injera  Biscuits  Other grains (rice, spaghetti, bread, barley)  Potatoes  Bovine or animal milk  Eggs | 26 (25.5%)  13 (12.7%)  9 (8.8%)  11 (10.8%)  24 (23.5%)  4 (3.9%) |
